# Supplementary material for: A yeast phenomic model for the influence of Warburg metabolism on genetic buffering of doxorubicin
Source: Cancer Metab. 2019 Oct 23;7:9. doi: 10.1186/s40170-019-0201-3 (PMC6806529; doi:10.1186/s40170-019-0201-3)
Supplement: Supplementary file 9 — Additional file 9. GO term-specific heatmaps for REMc/GTF-enriched clusters. GO term-specific heatmaps for significant GO process terms were generated as described in methods and Figs. 3 and 4. Any related child terms are presented in subsequent pages of the parent file name. GO terms with more than 100 children, with 2 or fewer genes annotated to the term, or a file size over 300KB are not shown. All heatmaps are generated with the same layout (see Figs. 3 and 4). [file 40170_2019_201_MOESM9_ESM.bz2 › Additional_File9_GOTermHeatmaps/Additional_File9_GOTermHeatmap/tRNA_wobble_base_modification.pdf]

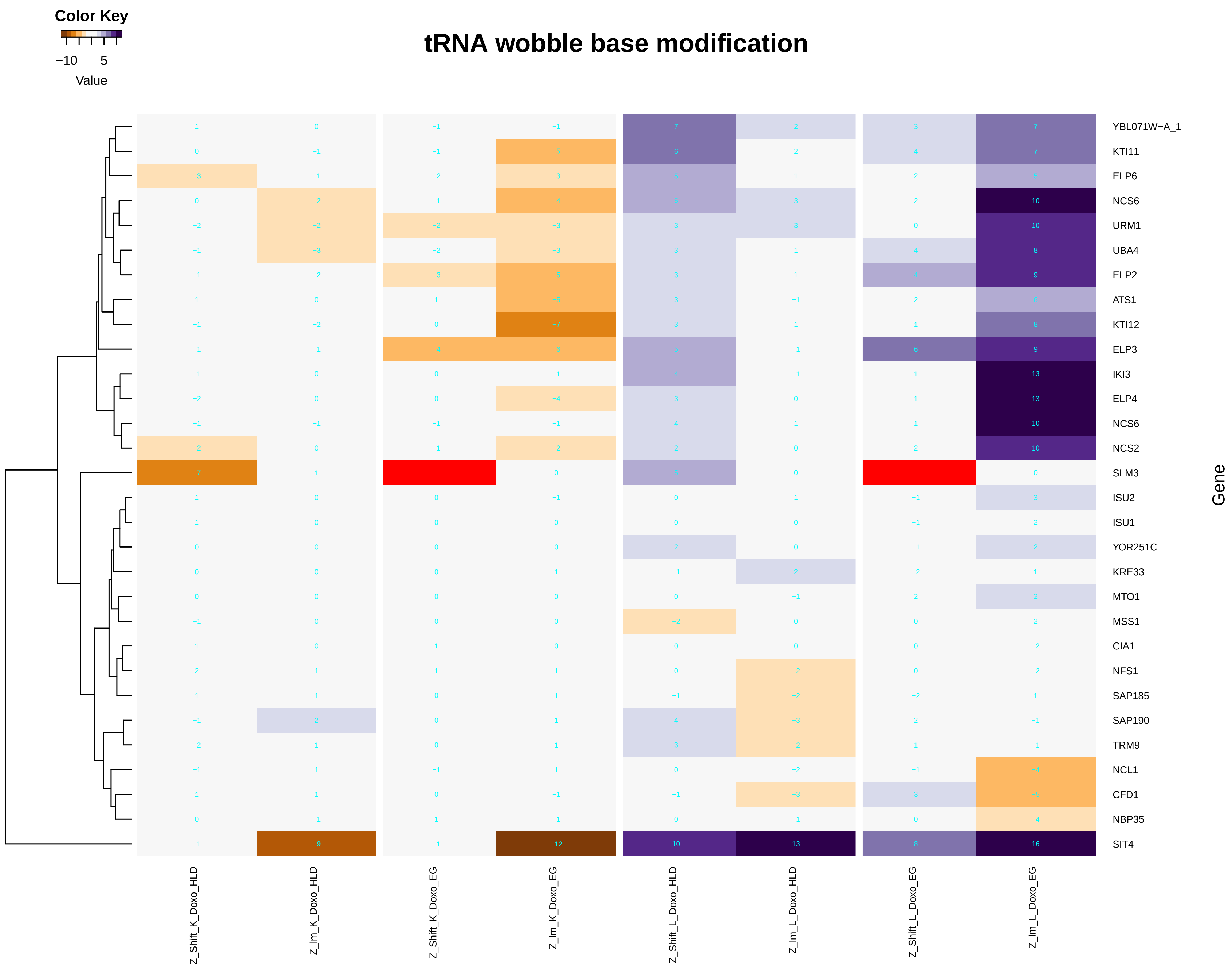
Color Key
−10
5
Value
tRNA wobble base modification
YBL071W−A_1
KTI11
ELP6
NCS6
URM1
UBA4
ELP2
ATS1
KTI12
ELP3
IKI3
ELP4
NCS6
NCS2
SLM3
ISU2
ISU1
YOR251C
KRE33
MTO1
MSS1
CIA1
NFS1
SAP185
SAP190
TRM9
NCL1
CFD1
NBP35
SIT4
Gene
Z_Shift_K_Doxo_HLD
Z_Im_K_Doxo_HLD
Z_Shift_K_Doxo_EG
Z_Im_K_Doxo_EG
Z_Shift_L_Doxo_HLD
Z_Im_L_Doxo_HLD
Z_Shift_L_Doxo_EG
Z_Im_L_Doxo_EG

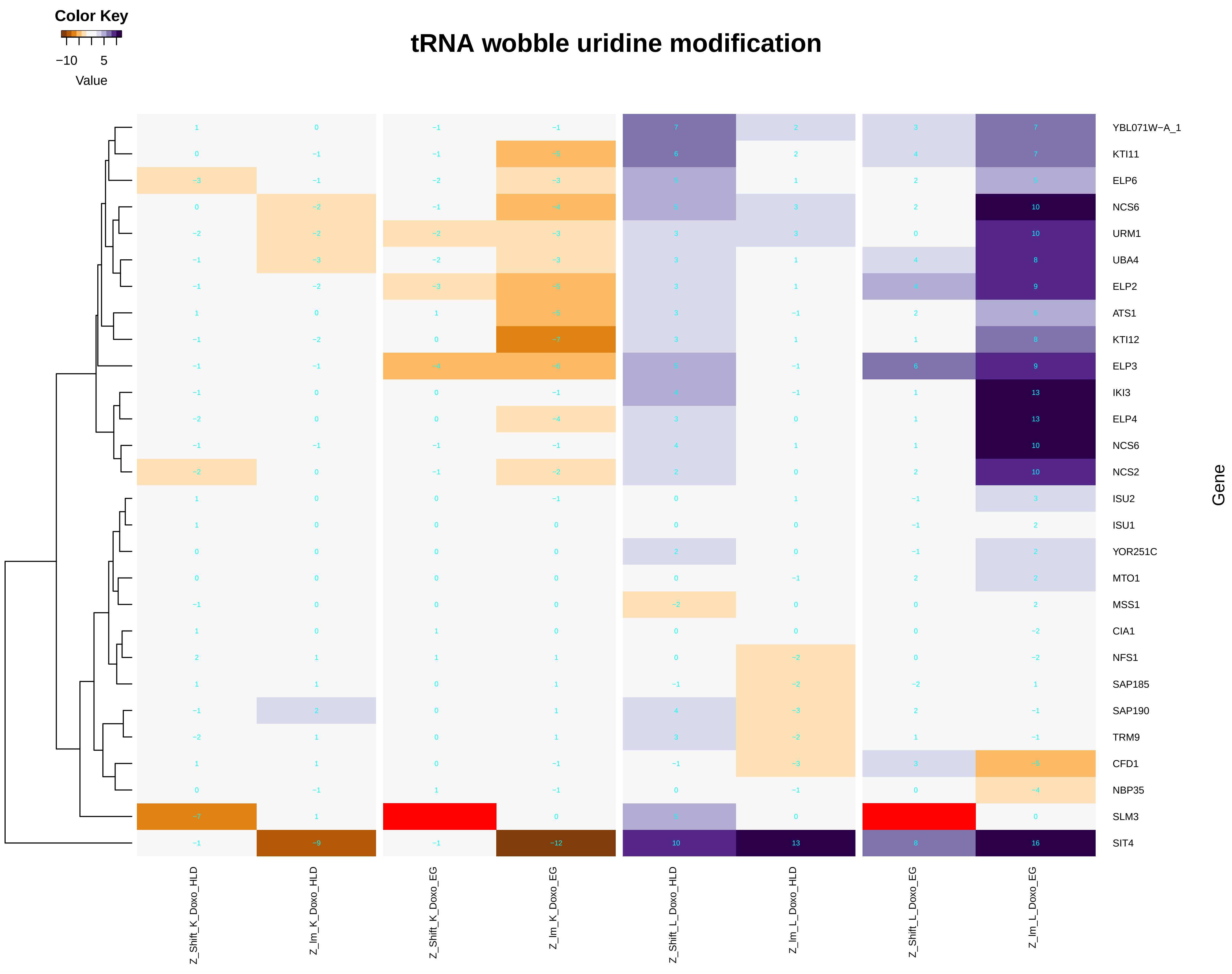
Color Key
−10 5
Value
tRNA wobble uridine modification
YBL071W−A_1
KTI11
ELP6
NCS6
URM1
UBA4
ELP2
ATS1
KTI12
ELP3
IKI3
ELP4
NCS6
NCS2
ISU2
ISU1
YOR251C
MTO1
MSS1
CIA1
NFS1
SAP185
SAP190
TRM9
CFD1
NBP35
SLM3
SIT4
Gene
Z_Shift_K_Doxo_HLD
Z_Im_K_Doxo_HLD
Z_Shift_K_Doxo_EG
Z_Im_K_Doxo_EG
Z_Shift_L_Doxo_HLD
Z_Im_L_Doxo_HLD
Z_Shift_L_Doxo_EG
Z_Im_L_Doxo_EG

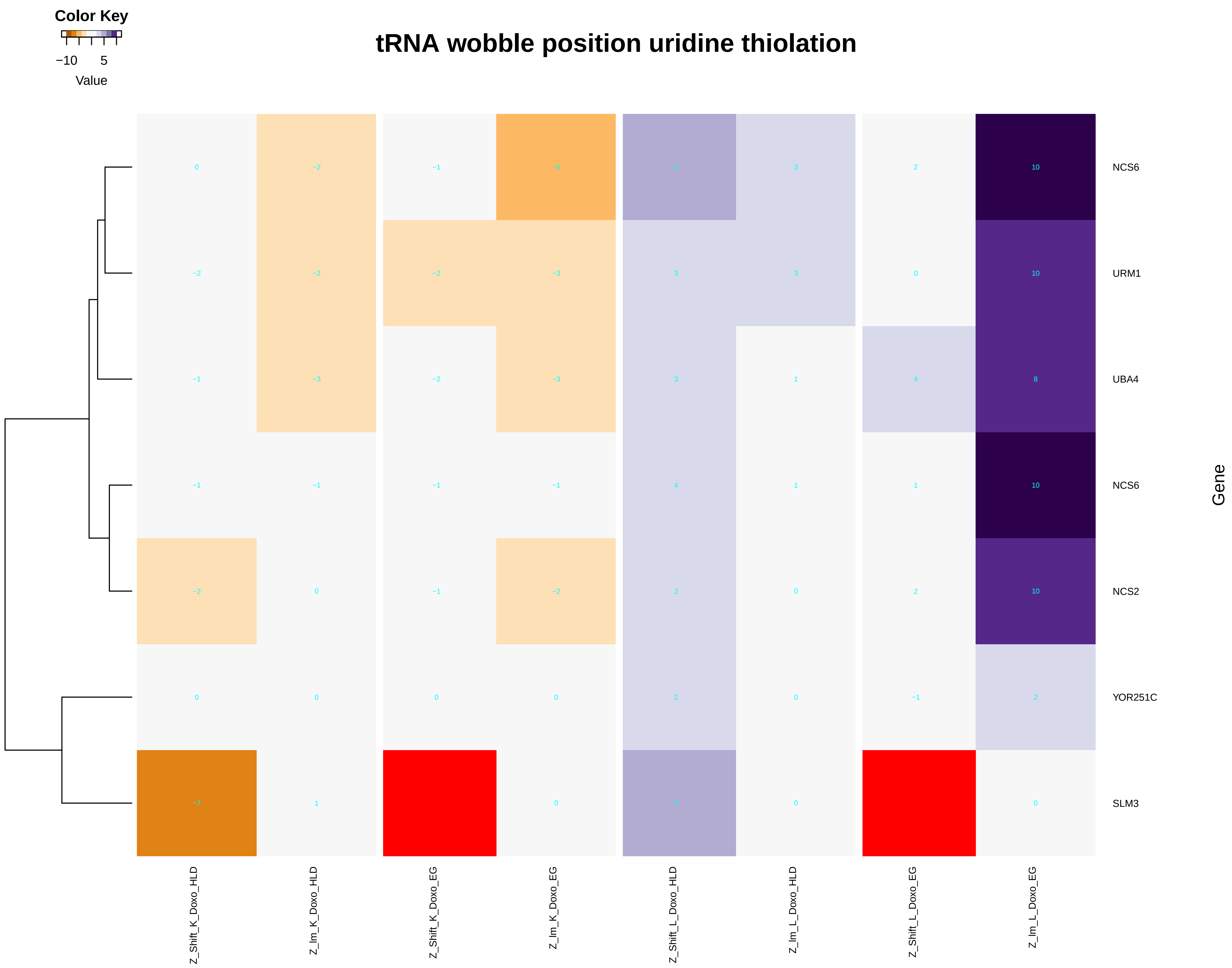
Color Key
−10
5
Value
tRNA wobble position uridine thiolation
NCS6
URM1
UBA4
NCS6
NCS2
YOR251C
SLM3
Gene
Z_Shift_K_Doxo_HLD
Z_Im_K_Doxo_HLD
Z_Shift_K_Doxo_EG
Z_Im_K_Doxo_EG
Z_Shift_L_Doxo_HLD
Z_Im_L_Doxo_HLD
Z_Shift_L_Doxo_EG
Z_Im_L_Doxo_EG

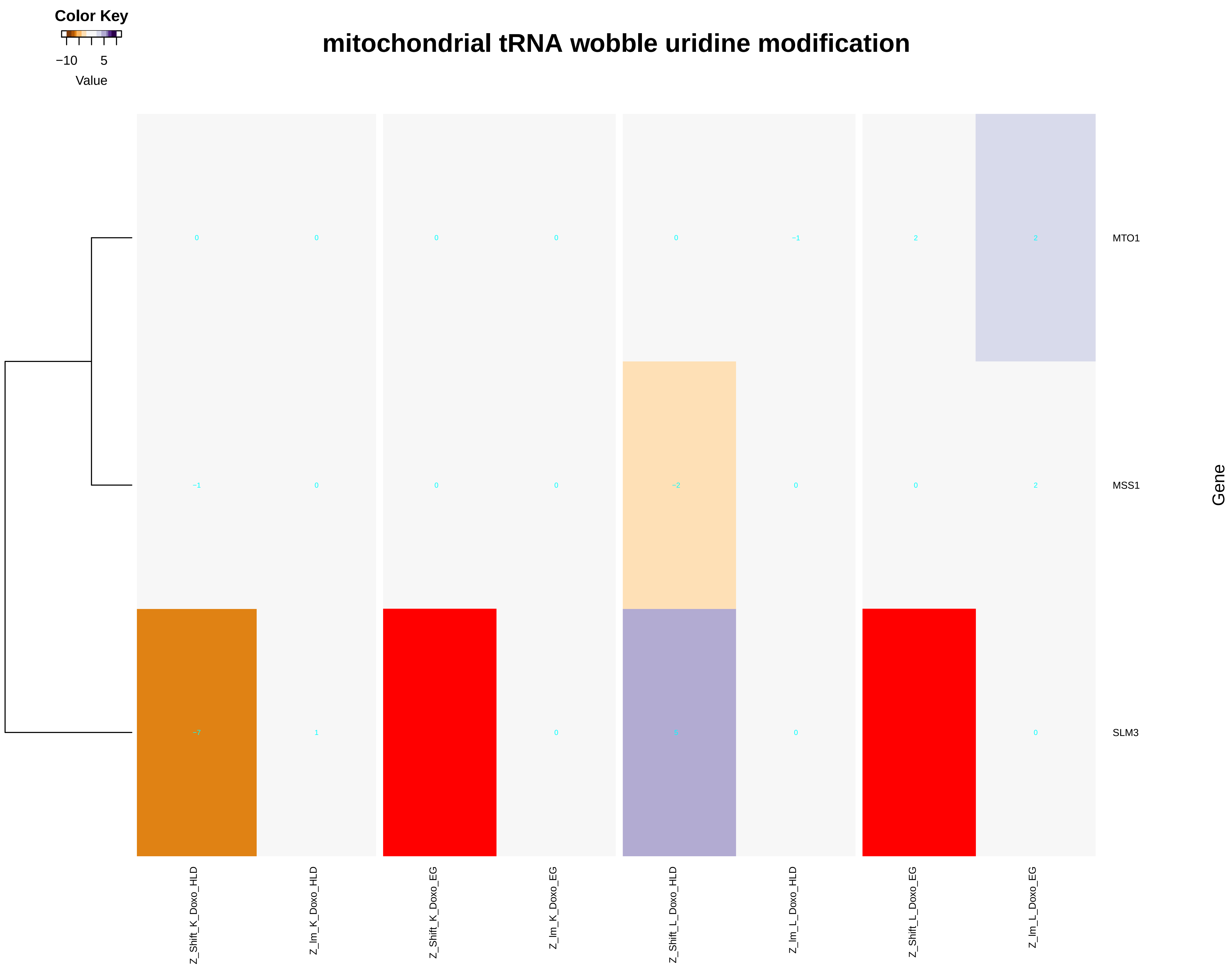

Color Key
−10
5
Value
mitochondrial tRNA wobble uridine modification
MTO1
MSS1
SLM3
Gene
Z_Shift_K_Doxo_HLD
Z_Im_K_Doxo_HLD
Z_Shift_K_Doxo_EG
Z_Im_K_Doxo_EG
Z_Shift_L_Doxo_HLD
Z_Im_L_Doxo_HLD
Z_Shift_L_Doxo_EG
Z_Im_L_Doxo_EG
